# Neuropilin-1 is a glial cell line-derived neurotrophic factor receptor in glioblastoma

**DOI:** 10.18632/oncotarget.18630

**Published:** 2017-06-27

**Authors:** Shen Sun, Yu Lei, Qi Li, Yue Wu, Lin Zhang, Pei-Pei Mu, Guang-Quan Ji, Chuan-Xi Tang, Yu-Qian Wang, Jian Gao, Jin Gao, Li Li, Lang Zhuo, Yun-Qing Li, Dian-Shuai Gao

**Affiliations:** ^1^ Department of Anatomy and Histology, The Fourth Military Medical University, Xi’an, Shanxi, China; ^2^ Department of Neurobiology and Anatomy, Xuzhou Key Laboratory of Neurobiology, Jiangsu Key Laboratory of New Drug Research and Clinical Pharmacy, Xuzhou Medical University, Xuzhou, Jiangsu, China; ^3^ Department of Histology and Embryology, Xuzhou Medical University, Xuzhou, Jiangsu, China; ^4^ Department of Neurobiology, School of Basic Medical Sciences, Shanghai Medical College of Fudan University, Shanghai, China; ^5^ Jiangsu Key Laboratory of New Drug Research and Clinical Pharmacy, Xuzhou Medical University, Xuzhou, Jiangsu, China; ^6^ Department of Pathophysiology, Xuzhou Medical University, Xuzhou, Jiangsu, China; ^7^ Department of Epidemiology, School of Public Health, Xuzhou Medical University, Xuzhou, Jiangsu, China

**Keywords:** glial cell line-derived neurotrophic factor, glioblastoma, membrane receptor, neuropilin-1, cell proliferation

## Abstract

The aim of this study was to identify the receptor for glial cell line-derived neurotrophic factor (GDNF) in glioblastoma multiforme (GBM). After GST pull-down assays, membrane proteins purified from C6 rat glioma cells were subjected to liquid chromatography-tandem mass spectrometry (LC-MS/MS). The differentially expressed proteins were annotated using Gene Ontology, and neuropilin-1 (NRP1) was identified as the putative GDNF receptor in glioma. NRP1 was more highly expressed in human GBM brains and C6 rat glioma cells than in normal human brains or primary rat astrocytes. Immunofluorescence staining showed that NRP1 was recruited to the membrane by GDNF, and NRP1 co-immunoprecipitated with GDNF. Using the NRP1 and GDNF protein structures to assess molecular docking in the ZDOCK server and visualization with the PyMOL Molecular Graphics System revealed 8 H-bonds and stable positive and negative electrostatic interactions between NRP1 and GDNF. RNAi knockdown of NRP1 reduced proliferation of C6 glioma cells when stimulated with GDNF. NRP1 was an independent risk factor for both survival and recurrence in GBM patients. High NRP1 mRNA expression correlated with shorter OS and DFS (OS: χ^2^=4.6720, *P*=0.0307; DFS: χ^2^=11.013, *P*=0.0009). NRP1 is thus a GDNF receptor in glioma cells and a potential therapeutic target.

## INTRODUCTION

Glioblastoma (GBM) is one of the most common primary tumors of the central nervous system (CNS) with poor survival outcomes. It is characterized by uncontrolled cellular proliferation that is driven by a complex integration of a various extracellular stimuli that signal through multiple membrane receptor systems, resulting in elaborate changes in gene expression [1]. Therefore, agents that block one or more aberrant signaling pathways that promote tumor growth and proliferation can potentially reduce the severe mortality and morbidity in GBM patients [2].

Glial cell line-derived neurotrophic factor (GDNF), a member of the GDNF family of ligands (GFL), is strongly expressed in human gliomas [3]. Knock-down of GDNF and its binding receptor, GDNF family receptor alpha 1 (GFRA1) decreases the proliferation of C6 glioma cells [4]. However, the mechanisms downstream of GDNF binding to C6 cells are not clear because of multiple signal transducing receptors for GDNF in different cells.

Since extracellular signaling molecules modulate cellular functions by binding to specific transmembrane receptor proteins [5], it is important to identify the GDNF receptors on glioma cells. GDNF transmits signals through a multi-component receptor system that consists of glycosylphosphatidyl inositol (GPI)-linked GFRA1 [6] and proto-oncogene tyrosine-protein kinase receptor RET [7]. Formation of the GDNF_2_–GFRA1_2_–RET_2_ heterohexameric complex results in transphosphorylation of RET, thereby activating the receptor tyrosine kinase signaling pathway [8–10]. However, binding of GDNF to the neural cell adhesion molecule, NCAM, instead of RET results in activation of Fyn, which regulates Schwann cell migration and axonal growth in hippocampal neurons [11]. In 2011, Bespalov *et al* reported that heparan sulfate proteoglycan, syndecan-3 (SDC3) was a novel receptor for GDNF, which either directly transmitted the GFL signals or acted as a co-receptor and presented GFLs to RET [12]. In many cases, ligands have multiple receptors, which can induce different responses in the same or different cell types [5]. Therefore, in view of the unique biological features of GBM, it is possible that hypersecretion of GDNF in combination with its signaling through multiple receptors plays a role in promoting GBM cell growth and proliferation [3].

In recent years, proteomics has helped identify novel protein-protein interactions (PPIs) [13]. Therefore, in this study, we used a combination of GST pull-down assays with mass spectrometry (MS) and bioinformatic methods to identify the membrane receptor for GDNF on rat C6 glioma cells.

## RESULTS

### GDNF promotes the proliferation of C6 glioma cells

Serum starvation of C6 glioma cells resulted in 80% G0/G1 phase cells compared to 63.3% in C6 cells grown with 10% FBS (*P*< 0.05). CCK-8 assay demonstrated that 40 ng/ml was the most optimal concentration of GDNF and the 48h time point showed peak C6 cell viability (***P < 0.001). These data were further confirmed by flow cytometry and EdU assays (**P< 0.01).

### NRP1 is the putative GDNF-receptor in glioma cells

**Figure 1:**
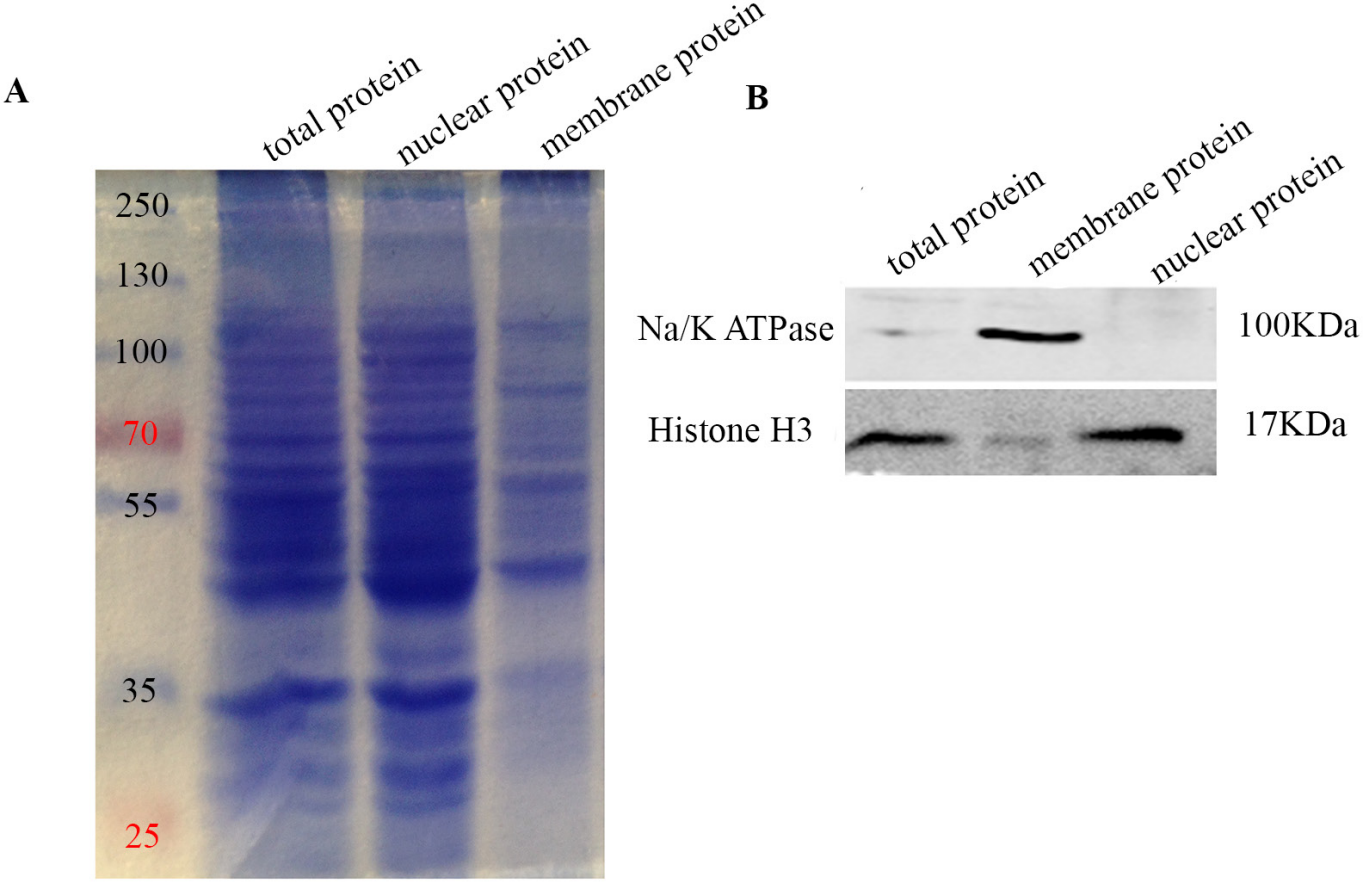
Western blot analysis of membrane proteins purification **(A)** Coomassie Brilliant Blue stained SDS-PAGE gel showing molecular weight marker (lane1), total C6 cellular protein (lane2), nuclear protein fraction (lane3) and purified membrane protein fraction (lane4). 30 μg protein was loaded in lanes 2-4. As shown, membrane fraction has fewer number of proteins compared to the nuclear fraction and total cellular protein. Molecular weight marker shows 250, 130, 100, 70, 55, 35, and 25kDa bands. **(B)** Western-blot analysis shows Na^+^/K^+^ ATPase (100 kDa membrane protein) and histone H3 (17 kDa nuclear protein) in total C6 cellular protein (lane1), nuclear protein fraction (lane2) and purified membrane protein fraction (lane3). As shown, Na^+^/K^+^ ATPase is enriched in the membrane fraction, whereas histone H3 is enriched in the nuclear fraction.

Membrane fractions were purified from rat C6 cells and primary astrocytes (ASTs) and separated on SDS-PAGE with nuclear fraction and total cellular protein and stained with Coomassie brilliant blue. Comparatively fewer membrane proteins were observed compared to total and nuclear fraction (Figure 1A). Western blot analysis showed that Na/K ATPase was present only in the membrane fraction and absent in the nuclear fraction demonstrating purity of the membrane protein preparation (Figure 1B).

**Figure 2:**
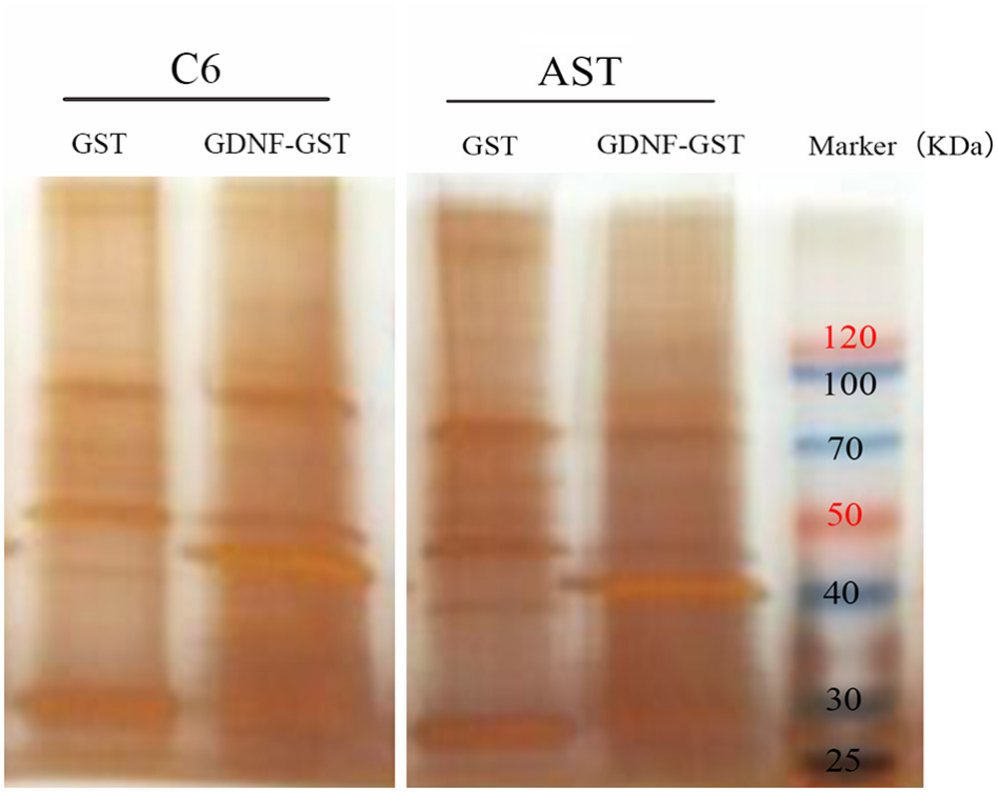
GST pull-down assay to identify membrane proteins binding to GDNF Silver stained SDS-PAGE gel shows membrane proteins from C6 rat glioma cells (C6) and primary rat astrocytes (AST) pulled down by either GST or GDNF-GST fusion protein. Lane1, C6-GST; Lane2, C6-GDNF-GST; Lane3, AST-GST; Lane 4, AST-GDNF-GST; Lane 5, protein molecular weight marker. Note: GST: GST alone; GDNF-GST: GDNF-GST fusion protein.

The membrane protein fractions from C6 rat glioma cells (C6) and primary rat astrocytes (AST) were then used for GST pull-down assay with GST or GST-GDNF fusion proteins and silver staining was performed after running the samples on SDS-PAGE (Figure 2). To identify the membrane proteins that bind to GDNF, liquid chromatography-tandem mass spectrometry (LC-MS/MS) results. More number of proteins and peptides were identified in the C6-GDNF-GST and AST-GDNF-GST groups than control GST alone groups (Table 1). Label-free quantitative (LFQ) method demonstrated that the distribution of protein abundance in all 4 groups was normal and there was higher correlation of protein abundance among the same group (data not shown).

**Table 1:** Summary of LC-MS/MS analysis

Samples	Total_number of spectra	Total number of identified_spectra	Total number of identified_peptides	Total number of identified_proteins
C6 GDNF-GST	49558	6445	4527	1652
C6 GST	48122	4770	3043	1231
AST GDNF-GST	48548	6934	4407	1537
AST GST	46530	4522	2978	1134

C6: C6 rat glioma cells; AST: primary rat astrocytes; GDNF-GST: fusion of GDNF and GST proteins; GST: GST protein alone.

The differentially expressed proteins (DEPs) identified by MS in the 4 groups were analyzed by bioinformatics. The strategy involved analyzing (1) membrane proteins that were bound to GDNF in the pull down assay; (2) those that were found in both C6-GDNF-GST and AST-GDNF-GST groups and; (3) those that demonstrated higher binding capacity in C6 compared to AST groups.

The DEPs were annotated with Gene Ontology (GO) terms using PANTHER Classification System with the search parameter ‘membrane receptor’ [14]. We selected and combined (1) receptor activity (GO:0004872) and receptor binding (GO:0005102) for molecular function (MF); (2) biological adhesion (GO:0022610) and growth (GO:0040007) for biological process (BP); (3) extracellular matrix (GO:0031012), extracellular region (GO:0005576), integral to membrane (GO:0016021) and plasma membrane (GO:0005886) for cellular component (CC); and (4) cell adhesion molecule (PC00069), extracellular matrix protein (PC00102), membrane traffic protein (PC00150), receptor (PC00197) and transmembrane receptor regulatory/adaptor protein (PC00226) for protein Class (PC). Based on this analysis, we identified Attractin (ATRN), neuropilin-2 (NRP2) and NRP1 as common proteins (data not shown).

**Figure 3:**
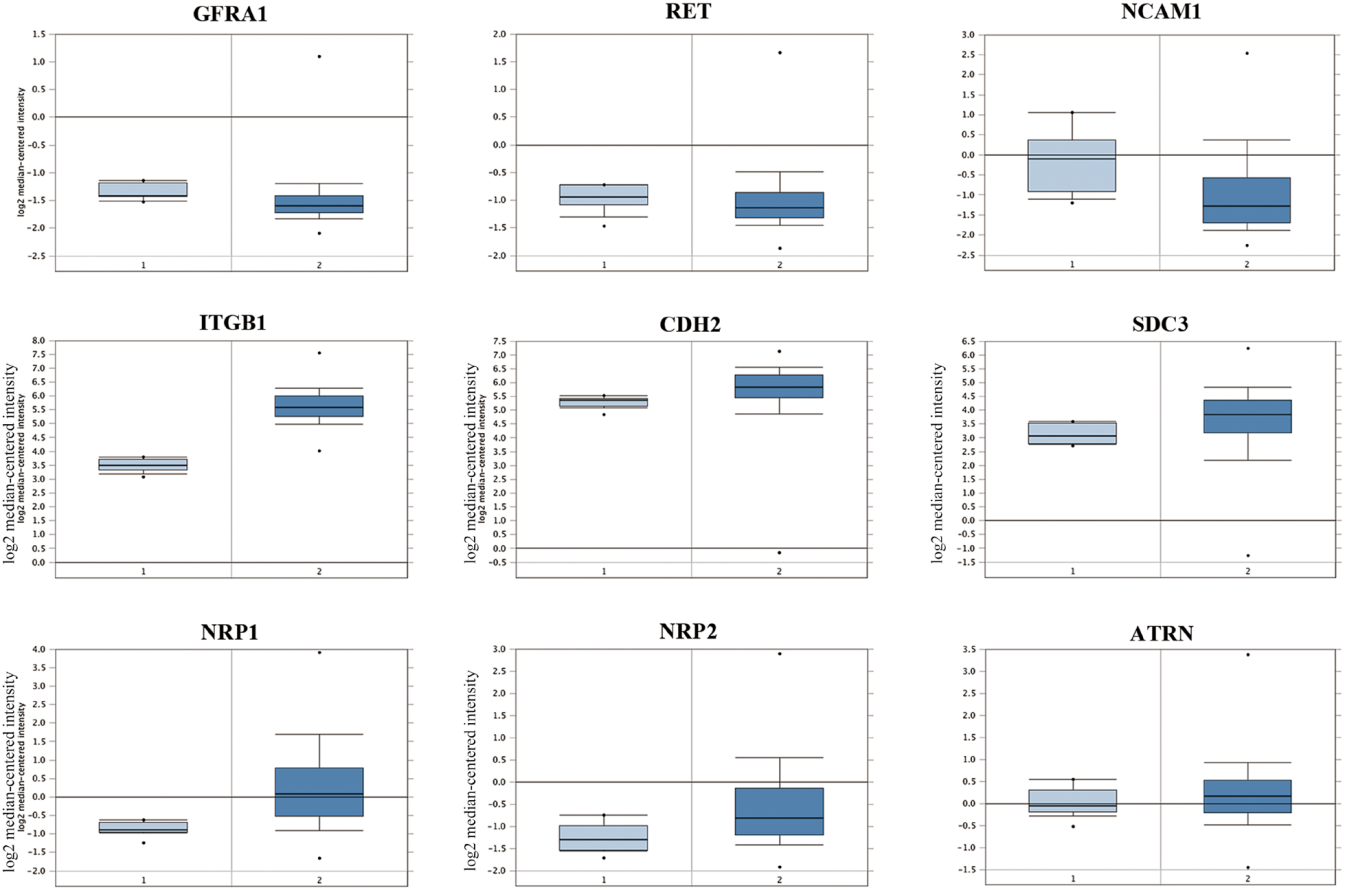
Analysis of candidate membrane receptors for GDNF in human glioblastoma patient samples Comparison of mRNA levels (log2 median-centered intensity) of human GFRA1, RET, NCAM1, ITGB1, CDH2, SDC3, NRP1, NRP2 and ATRN in (1) normal brain samples (total 10 samples) and (2) glioblastoma brain samples (542 samples) from TCGA Brain dataset analyzed by Oncomine^®^ Platform. Results showed that mRNA levels of GFRA1, RET, NCAM1, CDH2, SDC3 and ATRN in glioblastoma brain samples were similar to the normal brain group (P>0.05). On the other hand, mRNA levels of NRP1, NRP2 and ITGB1 were significantly higher in the glioblastoma brain samples compared to the normal brains (*P*<0.001 for NRP1; *P*<0.01 for NRP2 and ITGB1).

Analysis of human GBM and normal brain data from TCGA datasets revealed that mRNA levels of GFRA1, RET, NCAM1, CDH2, SDC3 and ATRN were comparable ( *p*>0.05), whereas mRNA levels of Integrin beta-1 (ITGB1), NRP1 and NRP2 were elevated in the GBM brains (*P*<0.01, FC>1.5; Figure 3).

### NRP1 is overexpressed in glioma cells and recruited to the membrane by GDNF

**Figure 4:**
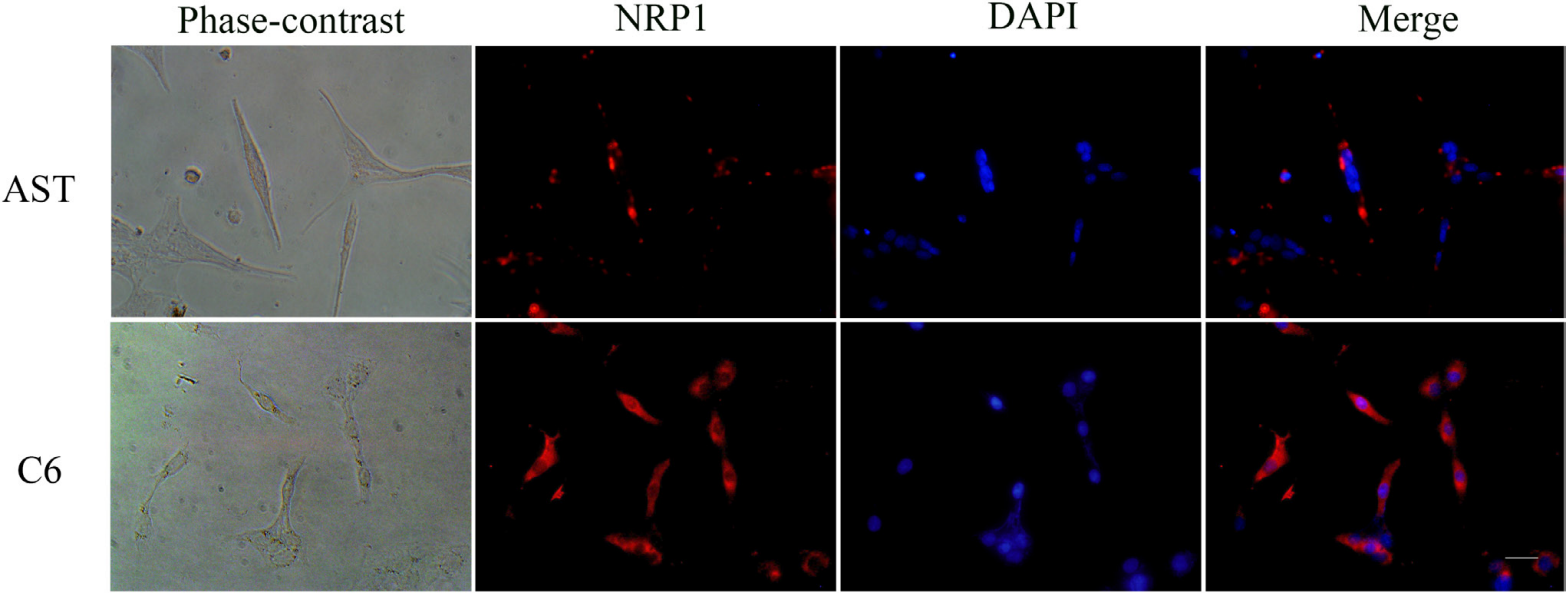
Immunofluorescence staining of NRP1 in C6 rat glioma cells and primary rat astrocytes Representative immunofluorescence staining images of C6 rat glioma cells (C6) and rat primary astrocytes (AST). (Left to right) Phase contrast, NRP1 staining (probed with anti-NRP1 antibody; red), nuclear staining (DAPI; blue) and Merged (NRP1 and DAPI) images are shown for AST (top) and C6 (bottom) cells. NRP1 expression was significantly higher in C6 cells than AST (P<0.05). Note: scale bar = 40 μm.

**Figure 5:**
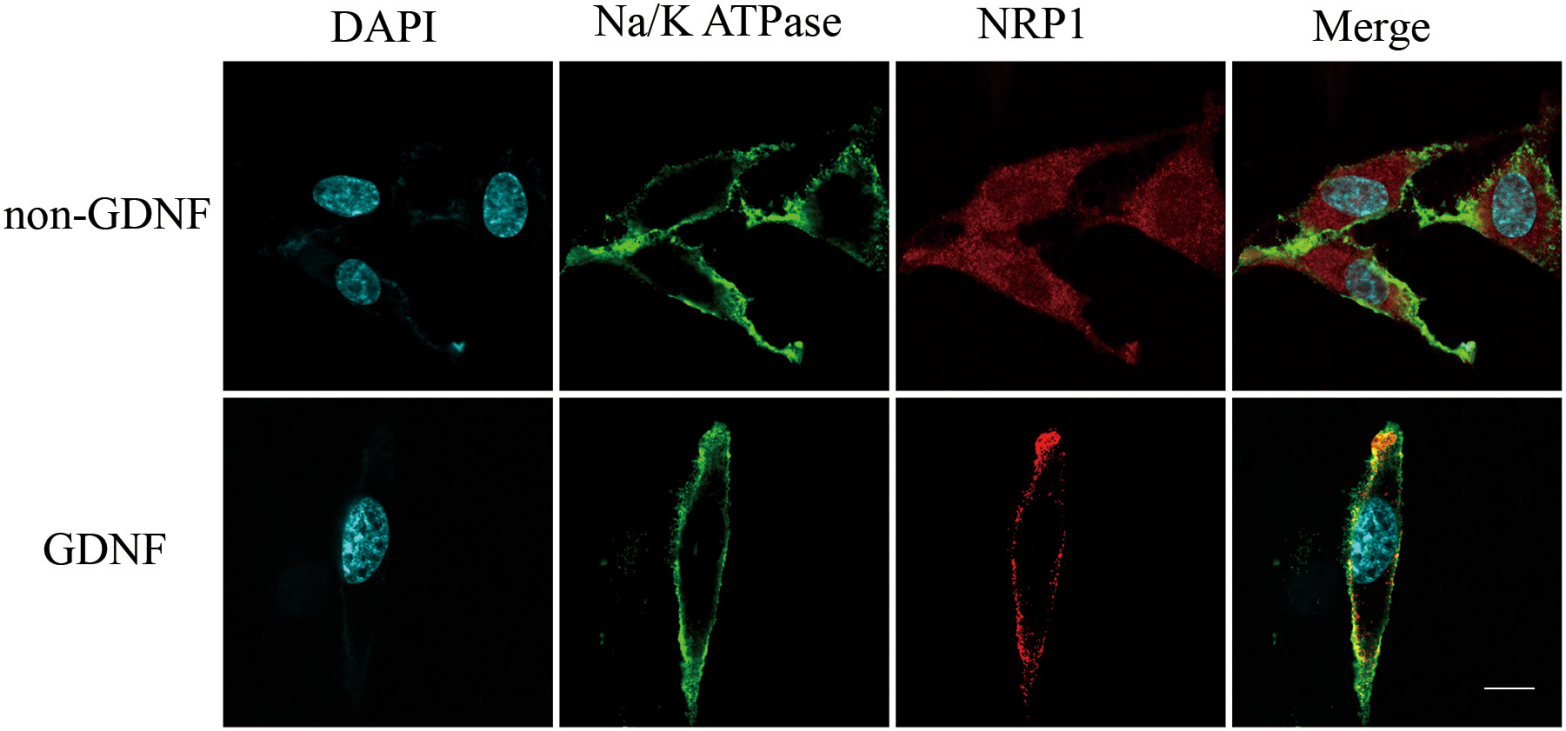
Immunofluorescence analysis showing membrane recruitment of NRP1 by exogenous GDNF in C6 glioma cells Representative laser confocal immunofluorescence images of C6 rat glioma cells stained with (left to right) DAPI (blue, nuclear staining), anti-Na^+^/K^+^ ATPase antibody (green, membrane staining), anti-NRP1 antibody (red) and merge (DAPI, anti-Na^+^/K^+^ ATPase and anti-NRP1) in GDNF treated (bottom) and non-GDNF treated (top) cells. NRP1 was recruited to the cell membrane after short-term GDNF treatment, whereas NRP1 was localized in the cytoplasm in the non-GDNF group. Note: scale bar = 20 μm.

NRP1 was in the top 1% among the highly expressed mRNAs in the GBM brains compared to normal brains from the TGCA dataset (Figure 3; *P*-value= 7.03E^-15^, t-Test=16.370, Fold Change = 2.205). Immunofluorescence staining also demonstrated high NRP1 expression in C6 glioma cells compared to normal astrocytes (Figure 4; p<0.05). Further, immunofluorescent (IF) staining showed that exogenous GDNF recruited more NRP1 to the cell membrane of C6 glioma cells, whereas the non-GDNF treated cells showed diffused cytoplasmic staining for NRP1 (Figure 5).

### Structural and functional analysis of NRP1 interaction with GDNF

**Figure 6:**
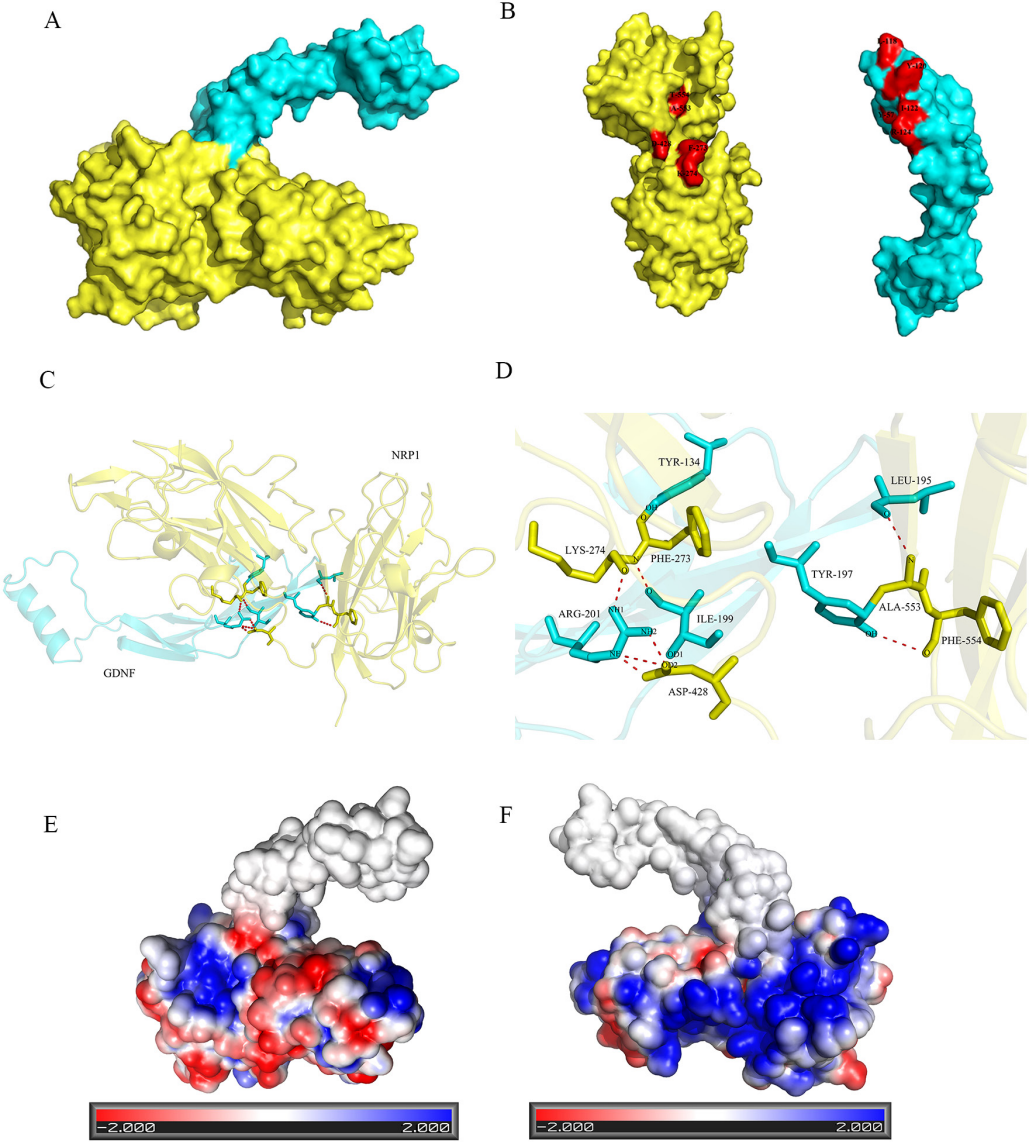
Illustration of binding interactions between GDNF and NRP1 Interaction between GDNF and NRP1 based on protein structure docking performed by the ZDOCK server and visualized by PyMOL Molecular Graphics System. **(A)** A surface presentation of the GDNF-NRP1 binding interface. Blue: GDNF; yellow: NRP1. **(B)** Surface images of GDNF (Blue) and NRP1 (yellow) showing H-bond between amino acid residues of GDNF and NRP1 (red). **(C)** A ribbon and stick presentation of GDNF-NRP1 binding. Blue: GDNF; yellow: NRP1. **(D)** A stick presentation of the GDNF-NRP1 binding interface showing the 8 H-bonds between the corresponding amino acids. **(E)** Surface presentation showing electrostatic interaction between NRP1 and GDNF on one side. Red denotes negative charge whereas blue denotes positive charge. **(F)** Surface presentation showing electrostatic interaction between NRP1 and GDNF on the opposite side to that shown in E.

The molecular docking between GDNF and NRP1 was performed with ZDOCK software and the molecular conformations with top 10 docking scores were selected (data not shown). Visual analysis by PyMOL software on the molecular conformation with the highest score (11201.180) showed formation of 8 hydrogen bonds (H-bonds) between GDNF and NRP1 (Figure 6A-6D and Table 2). Among them, aa at 201 (ARG) of GDNF formed 4 H-bonds with aa residues 428 (ASP) and 274 (LYS) of NRP1. Stable positive and negative electrostatic interactions were also demonstrated between NRP1 and GDNF by the Adaptive Poisson-Boltzmann Solver (APBS) plug-in of PyMOL software (Figure 6E and 6F). These data demonstrated the molecular interaction between NRP1 and GDNF on the surface of C6 glioma cells.

**Table 2:** Summary of hydrogen bonding between GDNF and NRP1

Hydrogen bond	NRP1			GDNF			distance (Å)
	AA No.	AA	Atom	AA No.	AA	Atom	
1	273	PHE	O	134	TYR	OH	1.43
2	554	PHE	O	197	TYR	OH	3.34
3	428	ASP	OD1	201	ARG	NE	3.29
4	428	ASP	OD2	201	ARG	NE	2.02
5	274	LYS	O	201	ARG	NH1	2.76
6	428	ASP	OD1	201	ARG	NH2	2.19
7	274	LYS	N	199	ILE	O	2.42
8	553	ALA	N	195	LEU	O	2.87

Note: AA: amino acid; O, OD1, OD2 and OH: oxygen; N, NE, NH1 and NH2: nitrogen;

Based on protein structure analysis of NRP1-GDNF binding by ZDOCK server and visualized by PyMOL Molecular Graphics System.

**Figure 7:**
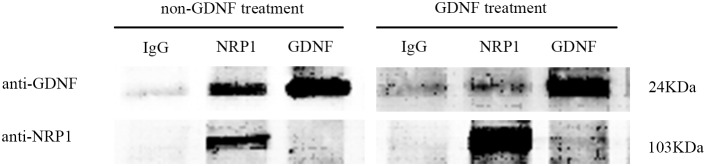
Co-immunoprecipitation analysis demonstrating NRP1 and GDNF binding Western blot analysis showing results of Co-IP of membrane proteins from non-GDNF and GDNF treated (40 ng/ml) groups with either IgG (negative control), anti-NRP1 or anti-GDNF antibodies. NRP1 (103kDa) binds to GDNF (24kDa) in both non-GDNF and GDNF treatment groups. As shown, NRP1 is enriched in the GDNF treated group.

To confirm the interaction between GDNF and NRP1, C6 cells were treated with or without exogenous GDNF and co-imunoprecipitated with either IgG (control group), anti-NRP1 (NRP1 group) or anti-GDNF (GDNF group). Western blot analysis showed that NRP1 was co-immunoprecipitated with GDNF in both groups; stoichiometric ratios of NRP1 and GDNF in both groups were very different (Figure 7).

### GDNF-NRP1 interaction promotes proliferation of C6 glioma cells

**Figure 8:**
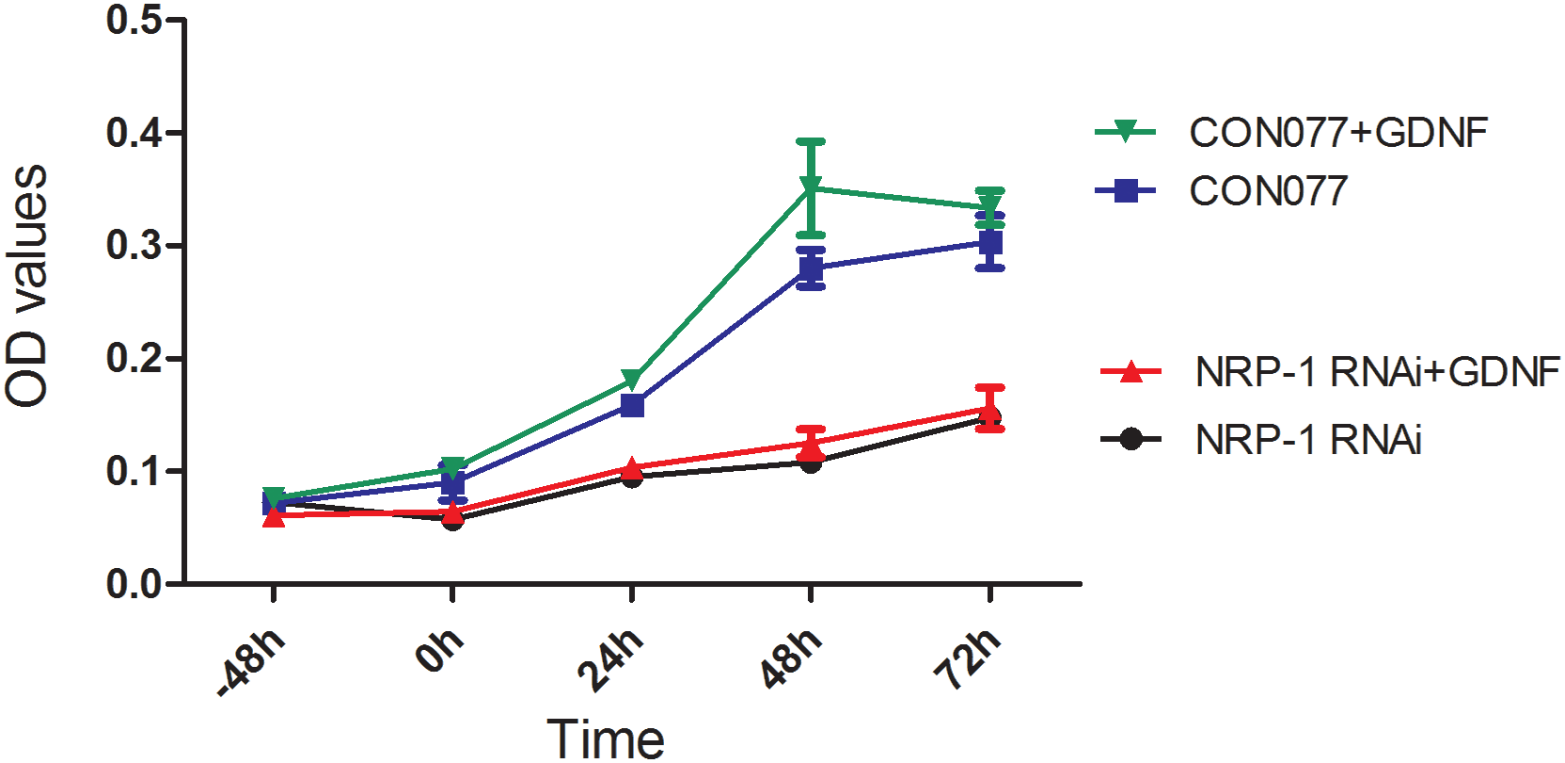
NRP1 RNAi reduces proliferation of GDNF-treated C6 rat glioma cells C6 rat glioma cells were infected with lentiviruses containing NRP1 shRNA or CON077 (control shRNA) for 48 h and then treated with or without exogenous GDNF. Plot shows CCK-8 assay measurements of cell proliferation at -48, 0, 24, 48 and 72h. As shown, NRP1 knockdown demonstrated lower OD values than the CON077 group at all time points after adding exogenous GDNF. Data were analyzed as mean ± SD from 3 replicate experiments.

Next, to ascertain if GDNF promoted proliferation of C6 glioma cells through NRP1, we performed knockdown of rat *NRP1* using the lentivector shRNA transduction of C6 glioma cells. CCK8 proliferation assay showed that *NRP1* RNAi resulted in decreasing proliferation of C6 glioma cells treated with exogenous GDNF compared to C6 cells transduced with CON77 RNAi (Figure 8; *P* < 0.05). This suggested that NRP1 interaction with exogenous GDNF promoted proliferation of C6 glioma cells.

### NRP1 expression correlates with GBM prognosis

**Figure 9:**
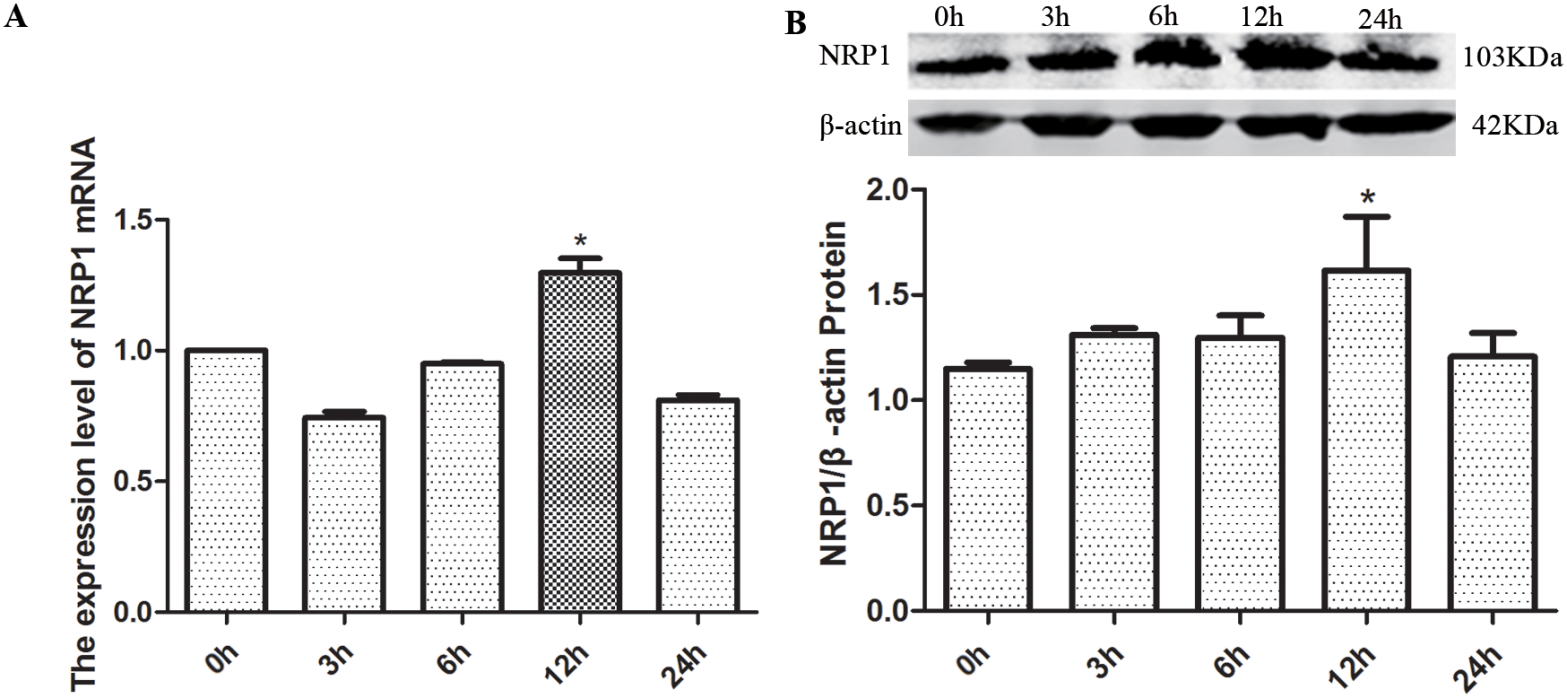
The effects of exogenous GDNF on NRP1 mRNA and protein expression in C6 cells **(A)** Histogram shows relative NRP1 mRNA levels in C6 cells at 0, 3, 6, 12 and 24h after treatment with 40 ng/ml GDNF. Relative levels plotted represent ratio of experiment group/control group. **(B)** Histogram shows quantification of relative NRP1 protein levels (ratio of NRP1/β-actin) in C6 cells at 0, 3, 6, 12 and 24h after treatment with 40 ng/ml GDNF. Representative western blot is also shown. The data represent the mean ± SEM of three independent experiments. *P < 0.05.

Treatment of C6 glioma cells with exogenous GDNF resulted in increased expression of NRP1 protein and mRNA (Figure 9). To decipher the clinical significance of this finding, the association of overall survival (OS) and disease-free survival (DFS) was analyzed with NRP1 mRNA levels in the TCGA GBM dataset. GBM patients with high NRP1 mRNA expression demonstrated shorter OS and DFS than patients with low or normal NRP1 mRNA levels (OS: χ^2^=4.6720, *P*=0.0307, Table 3; DFS: χ^2^=11.013, *P*=0.0009, Table 4).

**Table 3:** Overall survival (OS) of high and low NRP1 expressing GBM patients

Group	N	mean	sd	p25	p50	p75
0	149	14.569	13.111	5.390	12.550	17.870
1	16	8.120	4.031	4.580	8.215	10.825
Total	165	13.944	12.660	5.160	11.830	17.480

Note: group 0: low or normal NRP1 expressing GBM patient group; 1: high NRP1 expressing GBM patient group.

N: samples number; sd: standard deviation; p25, p50 and p75: percentiles.

**Table 4:** Disease-free survival (DFS) of high and low NRP1 expressing GBM patients

Group	N	mean	sd	p25	p50	p75
0	104	10.178	11.574	4.385	6.375	12.745
1	15	3.749	2.351	2.300	2.890	4.760
Total	119	9.367	11.054	3.190	5.850	10.940

Note: group 0: low or normal NRP1 expressing GBM patient group; 1: high NRP1 expressing GBM patient group.

N: samples number; sd: standard deviation; p25, p50 and p75: percentiles.

**Figure 10:**
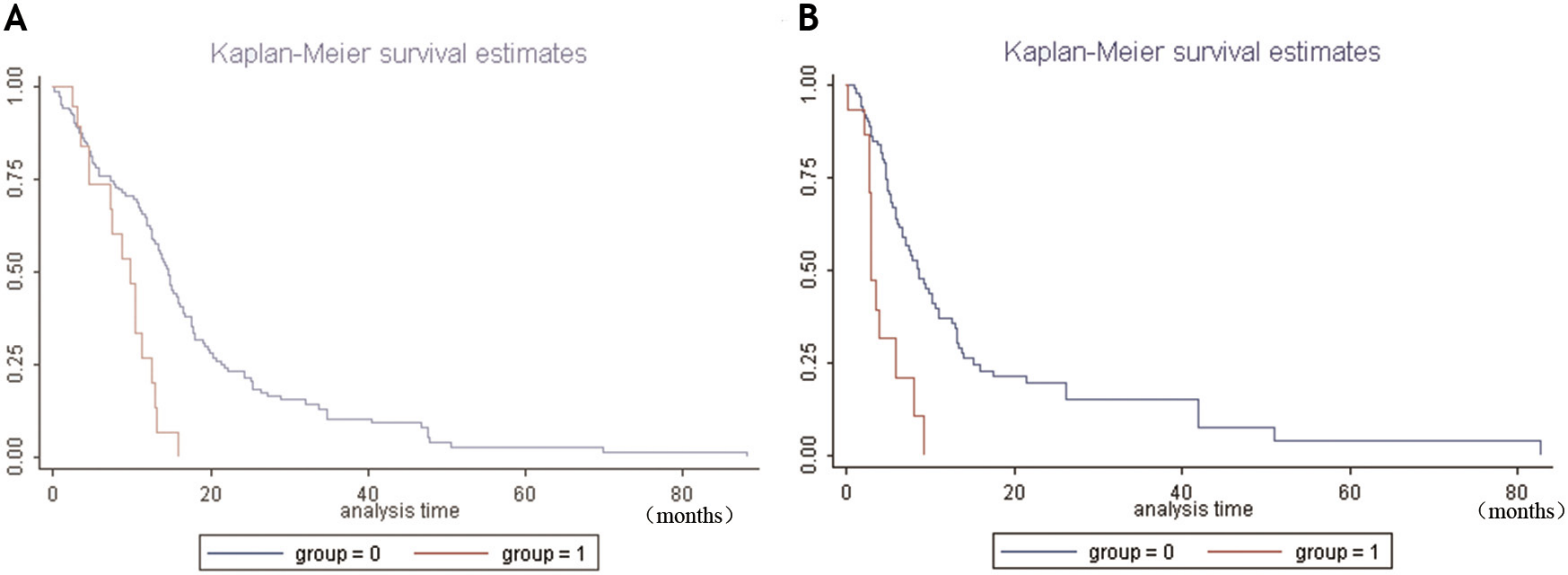
Kaplan-Meier survival analysis of GBM patients with high and low NRP1 mRNA levels **(A)** Kaplan-Meier curves for overall survival showing high NRP1 mRNA expressing GBM patient group (group 1) and normal NRP1 expressing GBM patient group (group 0). **(B)** Kaplan-Meier curves for disease-free survival showing high NRP1 mRNA expressing GBM patient group (group 1) and normal NRP1 expressing GBM patient group (group 0). Note: abscissa: survival months of patients; ordinate: survival rate; *P*< 0.05.

Multivariate proportional hazard model analysis demonstrated that high NRP1 mRNA expression was correlated with OS (Log likelihood =-527.61204, χ^2^=22.33, *P*=0.0001) and DFS (Log likelihood=-322.120, χ^2^=15.760, *P*=0.0239). When compared with age and gender, high NRP1 mRNA levels were an independent risk factor, reducing the survival time of patients and enhancing the risk of death by 2.5866 times compared to the control group (Table 5). The high NRP1 mRNA levels were also an independent risk factor for the recurrence of patients, with the risk of recurrence in the high NRP1 expression group being 3.6364 times higher than the control group (Table 6). Kaplan-Meier survival analysis demonstrated that higher NRP1 mRNA correlated with shorter OS and DFS (Figure 10A and 10B).

**Table 5:** Multivariate analysis of OS in comparison to high NRP1 levels

OS	Coeff.	Std. Err.	z	P>z	[95% CI]
Group	0.950	0.298	3.190	0.001	0.366-1.534
age	0.025	0.007	3.440	0.001	0.011-0.039
Gender	0.032	0.185	0.180	0.861	-0.330-0.395

Note: OS:overall survival; CI: confidence interval; Coeff: coefficient; Std. Err: standard error; z: z-score.

**Table 6:** Multivariate analysis of DFS in comparison to high NRP1 levels

DFS	Coeff.	Std. Err.	z	P>z	[95% CI]
Group	1.291	0.338	3.820	0.000	0.629-1.954
Age	0.014	0.009	1.700	0.089	-0.002-0.031
Gender	0.211	0.245	0.860	0.387	-0.268-0.691

Note: DFS:disease-free survival; CI: confidence interval; Coeff: coefficient; Std. Err: standard error;; z: z-score.

## DISCUSSION

In this study we demonstrated that NRP1 was the binding receptor in GBM cells with therapeutic potential. In previous studies, we had shown that trans-membrane proteins such as integrinβ1 [15], NCAM [16] and N-cadherin [17] were involved in protective effects of GDNF on dopaminergic neurons (DAs). Since GDNF is secreted in high amounts by gliomas, we postulated that GDNF membrane receptors would also be highly expressed in gliomas. Therefore, we compared the expression of GFRA1, RET, NCAM1, ITGB1, CDH2 and SDC3 between GBM and normal brains in the TCGA database using the Oncomine^®^ Platform. Among these, only ITGB1 showed high expression in GBM patient brains compared to normal brains.

Therefore, to identify the membrane receptors for GDNF in GBM, we perfomed a GST pull-down experiment in combination with LC-MS/MS and analyzed the membrane proteins binding to GDNF in C6 glioma cells and normal primary astrocytes, respectively. We found NCAM1 as the only differentially expressed protein (DEP) whereas the other known GDNF receptors including ITGB1 were not among the DEPs. GO analysis on the list of the DEPs identified by mass spectrometry using the PANTHER Classification System showed that 3 cell adhesion molecules, NRP1, NRP2 and ATRN, were putative GDNF receptors in glioma cells with roles in biological adhesion and growth. The Oncomine^®^ Platform analysis showed that NRP1 and NRP2 mRNA expression was increased in GBM brains compared to normal brains, whereas ATRN was similar. Hence, NRP1 and NRP2 were identified as candidate special receptor of GDNF in C6 glioma cells and we chose to further investigate NRP1 in this study.

Previous studies showed that R/KXXR/K was the C-terminal sequence for NRP1-binding proteins or peptides [18], with either arginine or lysine residues at the ends as found in such VEGF_165_[19, 20] and LD22-4 [19]. The isoform 1 of GDNF, which was chosen as the “canonical” sequence had a C-terminal sequence as “RKHSAKRCGCI” from amino acids 201 to 211, similar to R/KXXR/K consensus sequence. Molecular docking experiments demonstrated that amino acid 201 (ARG) of GDNF formed 4 H-bonds with amino acids 428 (ASP) and 274 (LYS) of NRP1, consistent with the expected C-terminal sequence RKHSAKRCGC (amino acids 201-210) of GDNF interacting with the extracellular domain of NRP1. Co-immunoprecipitation experiments established that NRP1 interacted with GDNF in C6 cells.

Neuropilins (NRPs) are non-tyrosine kinase transmembrane proteins with two identified homologues, NRP1 and NRP2 [21]. NRP1 and NRP2 share 47% homology and are located on chromosome l0pl2 and 2q34, respectively. Both of them are composed of three domains, extracellular, transmembrane and intracellular. NRP1 plays an important role in the normal development of the cardiovascular and nervous system [22]. Takagi *et al* reported that NRP1 mRNAs were present in the optic ganglion cells and absent in non-neuronal cells in the central and peripheral nervous system [23]. In recent years, NRP1 overexpression has been reported in many diseases [24–26] including cancers [27, 28].

In glioma, increased NRP1 expression is observed in endothelial cells and the neoplastic astrocytes of GBM [29]. NRP1 overexpression is also reported in glioma cell lines, C6, U251 and U87 [30]. Immunofluorescence staining demonstrated higher NRP1 expression on the membrane of C6 glioma cells than normal rat astrocytes. Also, higher NRP1 mRNA levels were observed in human GBM brain samples compared to normal in the TGCA GBM dataset.

NRP1 mediates progression of a variety of tumors including gliomas [31, 32]. It mediates the angiogenic effect of VEGF to provide nutrients for tumor growth [33, 34]. In human glioma cells, VEGF-VEGFR2-NRP1 signaling promotes the growth of tumors [35, 36]. In GBM, semaphorin3A (Sema3A)-NRP1 signaling mediates the invasion of cancer cells [37]. In U87MG glioma cells and vascular endothelial cells stimulated by hepatocyte growth factor, platelet-derived growth factor and VEGF, the intracellular domain of NRP1 induces tyrosine phosphorylation of p130^Cas^, which stimulates growth and invasion of gliomas [38–40].

Interestingly, GDNF and/or its putative receptor RET/NCAM crosstalk with the Semaphorins/VEGF family, which are the putative ligands of NRP1. Samaphorin3B interacts with GDNF signaling via NCAM to regulate axon guidance [41]. Sema4C-Plexin B2 signaling interacts with GDNF-RET signaling to regulate ureteric branching [42]. SEMA3A competes with VEGF165 for NRP1 binding and impairs GDNF signaling, whereas GDNF competes with VEGF-A signaling to inhibit ureteric bud branching morphogenesis [43]. GDNF also interacts with VEGF-VEGFR1 to increase human colon cancer cell motility [44]. VEGF-A/VEGFR2 cooperatively interacts with GDNF/RET signaling to induce ureteric bud cell proliferation and branching morphogenesis [45]. This suggests that NRP1, a putative receptor for Semaphorins/VEGF family, is a potential GDNF receptor.

Using laser scanning confocal microscopy, we observed that exogenous GDNF recruited NRP1 protein to the C6 glioma cell membrane. Coincidentally, stimulation of GDNF recruited RET to the lipid rafts resulting in RET/Src association [46], and protecting RET from proteasomal degradation [47]. This dynamic event was required for effective GDNF signaling. Therefore, we postulate that the recruitment of NRP1 to the C6 glioma cell membrane is necessary for effective GDNF signaling.

We observed decreased proliferation in GDNF-stimulated C6 glioma cells when *NRP1* was knocked down. Further, exogenous GDNF increased the expression of NRP1 mRNA and protein. H Osada *et al.* reported that the overexpression of NRP1 gene predicted tumor progression and poor prognosis in glioma [48]. We analyzed the association of NRP1 mRNA levels with overall survival and disease-free survival of GBM patients in the TCGA database by multivariate proportional hazard model. Results showed that NRP1 was an independent risk factor for GBM. Also, high NRP1 levels were associated with poor OS and DFS as well as early recurrence in GBM patients. This suggests that GDNF-NRP1 signaling promotes GBM progression and results in poor prognosis.

In conclusion, we demonstrated that NRP1 was the binding receptor of GDNF in glioma cells with therapeutic potential.

## MATERIALS AND METHODS

### C6 glioma cell line and primary astrocytes cultures

The rat C6 glioma cell line (Chinese Academy of Sciences, Shanghai, China) was grown in DMEM/F-12 medium (Gibco, Carlsbad, CA, USA) supplemented with 10% fetal bovine serum (FBS, Gibco, Carlsbad, CA, USA), 100U/ml penicillin (Vicmed, Jiangsu, China) and 0.1 mg/ml streptomycin (Vicmed, Jiangsu, China) at 37°C and 5%CO_2_.

The primary astrocytes were prepared from the cerebral cortex of ∼3 day old newborn Sprague-Dawley (SD) rats using the Neural Tissue Dissociation Kit (NTDK; MiltenyiBiotec Inc., BergischGladbach, Germany) [49]. After microglia and oligodendrocytes were removed, the rest of the cells were stained with the primary rabbit anti-glial fibrillary acidic protein (GFAP) antibody (Cappel, 1:100) followed by the secondary antibody, Alexa Fluor488 – conjugated Affinipure goat anti-rabbit (Vicmed, Jiangsu, China, 1:200) and observed under the fluorescent microscope to confirm the presence of primary astrocytes. The isolated primary astrocytes were then cultured in DMEM/F-12 medium containing 10% FBS at 37°C and 5% CO_2_.

### Cell cycle analysis by flow cytometry

Seven sets of C6 rat glioma cells were cultured in DMEM/F-12 medium containing 10% FBS at 37°C with 5% CO_2_ for 24h in 100mm culture dishes. Then, 6 sets were rinsed by PBS and grown in DMEM/F12 with 0.1% FBS (serum starvation medium), whereas 1 control set was grown in DMEM/F12 with 10% FBS for 1-6 days. The cells were then fixed in anhydrous ethanol at 4°C overnight, washed with PBS and incubated with 100 μg/ml RNAase (Tiangen Biotech, Beijing, China) in 500 μl PBS at 37°C for 30 min. The cells were then stained with 100 μg/ml propidium iodide (PI; Sigma, cat# P-4170) at 4°C for 30min and analyzed by flow cytometry (FACSCalibur, BD Biosciences, San Jose, CA, USA).

To determine the effects of GDNF on cell cycle, C6 glioma cells were cultured in a 60mm dish for 24h, and then starved for 48h. Then, the cells were treated with different concentrations of GDNF (0, 20, 40, 80, and 100 ng/ml) and further cultured for 2 days followed by PI staining and FACS analysis as described above. Modfit software (Verity, Topsham, ME) was used to determine the percentage of G0/G1 and S phase cells.

### CCK8 cell viability assay

The CCK-8 assay was used to determine the optimal GDNF concentration for maximal proliferation of C6 glioma cells. Briefly, the C6 glioma cells were cultured in 96-well plates for 24h and then starved for 48h. Then, they were treated with various concentrations of GDNF (0, 0.1, 1, 10, 20, 40, 80, 100, 200 and 400 ng/ml) and grown for 24, 48 or 72h (one plate was used at each time point with six replicates for each concentration). Then the cell viability assay was performed with the cell counting kit-8 assay kit (CCK-8; Dojindo Laboratories, Shanghai, China). The OD values were measured at 450 nm with a microplate reader (QuantBioTek Instruments, Winooski, VT, USA).

### EdU cell proliferation assay

We performed the EdU assay using Cell Light EdU DNA imaging Kit (RiboBio Co., Ltd. Guangzhou, China) to analyze cell proliferation. C6 glioma cells were incubated with EdU for 24h after appropriate treatments with different concentrations of GDNF. The percentage of EdU^+^ C6 cells was determined from the images.

### Total, membrane and nuclear proteins extraction

Total proteins extraction: Cells were collected, washed with cold PBS and centrifuged at 3000 rpm for 5 minutes. Then, the centrifugal precipitation was permeabilized by Radio Immunoprecipitation Assay (RIPA) lysis buffer (50mM Tris (pH 7.4), 150 mM NaCl, 1% Triton X-100, 1% sodium deoxycholate, 0.1% SDS, sodium orthovanadate, sodium fluoride, EDTA and leupeptin; Beyotime, China) and Phenylmethanesulfonyl fluoride (PMSF) (100:1), and further incubated on the ice for 30 minutes (vertexing every 5 minutes) at 4°C. Finally, after centrifugation at 12,000 rpm for 30 min at 4°C and removing the supernatant, the precipitation (total proteins) were transfered to a new tube and stored at -80°C.

Preparation of nuclear fractions: The nuclear proteins were prepared according to instructions of Nuclear and Cytoplasmic Protein Extraction Kit (Beyotime, China, P0027). Briefly, the cells were scraped with cold PBS and resuspended in buffer A with PMSF (100:1), vortexed and incubated on the ice for 10 min. Then the pellet was incubated with buffer B for 1 min. Further, after centrifugation at 16000 rpm for 5 min at 4°C, the supernatant (cytoplasmic fraction) was removed and the pellet was resuspended in nuclear extraction buffer C with PMSF (100:1) for 30 min (vertexing every 5 minutes) at 4°C, Finally, after centrifugation at 16,000 rpm for 10 min at 4°C, the supernatant (nuclear fraction) was stored at –80°C. At the same time, the extracted proteins were analyzed by western blot using rabbit anti-Histone H3 antibody (BS1660, bioworld, US, 1:500) to confirm the purity of the nuclear fractions.

Membrane proteins extraction: Membrane proteins were extracted from ∼0.5g C6 glioma cells and 0.2g normal primary astrocytes using the Mem-PER™ Eukaryotic Membrane Protein Extraction Kit (89826, Thermo Scientific, USA) according to manufacturer’s instructions. The proteins were quantitated. The purity of membrane fractions was determined by western blot using mouse anti-alpha 1 Sodium Potassium ATPase (Na/K-ATPase, ab7671, Abcam, 1:250).

### GST pull-down assay

The membrane proteins from rat C6 glioma cells (C6) or primary astrocytes (AST) were resuspended with 400 μl PBS and divided into 2 groups. Among them, 300 μl was mixed with 15 μg glutathione S-transferase tagged GDNF (GST-GDNF) and 100 μl was mixed with 5 μg GST alone. The 4 different groups, namely, C6 GDNF-GST, C6 GST, AST GDNF-GST and AST GST were gently shaken for 1h at 4°C. Then, 60 μl and 20 μl GST beads (Promega, V8611, USA) were added to the groups with GST-GDNF and GST, respectively, followed by overnight shaking at 4°C. Then, the 4 groups were cleaned thrice with PBS and eluted with 60 μl and 20 μl elution buffer (50mM glutathione in 50mM Tris, pH8.1), respectively.

The eluted samples were boiled in SDS-PAGE loading buffer and 12% SDS-PAGE was run at 90 V for 20 min. When loading buffer in the samples had run to at least 1/3^rd^ of the sepration gel, according to the instructions for the Fast Silver Stain Kit (P0017S, Beyotime, China), the gel was immersed successively in fixation fluid (50% ethanol, 10% acetic acid and 40% DDH_2_O), 30% ethanol, DDH_2_O, 100ml sensitizer solution, DDH_2_O, 100ml silver stain solution, DDH_2_O, silver color-substrate solution, silver stop buffer and DDH_2_O. All the steps were carried out with constant shaking at room temperature for several minutes. Finally, the silver stained gels were stored in DDH_2_O.

### Liquid chromatography-tandem mass spectrometry

The proteins were in-gel digested by 0.01 μg/μl trypsin at 37°C overnight using the filter aided sample preparation (FASP) technique. Peptide liquid phase separation was performed with the Prominence nano (LC-20AD, SHIMADZU) and processed by electrospray ionization (ESI) in the Q-EXACTIVE (QE) mass spectrometer (Thermo Fisher Scientific, San Jose, CA). The mass spectral files were analyzed using the Uniprot_rat database (http://www.uniprot.org/) by the Proteome Discoverer™ Software 1.4 (Thermo Fisher Scientific). Finally, the protein identification results were verified by analyzing the distribution of peptides, peptide charge and coverage, protein abundance and abundance correlation between samples.

### Bioinformatics analysis

The differentially expressed proteins (DEPs) of C6 rat glioma cells and primary rat astrocytes, identified from the MS, were compared using the GO database of the PANTHER Classification System [50] to identify the potential glioma specific receptors for GDNF.

### Immunofluorescence & confocal miroscopy

C6 and primary rat astrocytes were washed thrice with PBS and fixed with 4% paraformaldehyde for 45 min. Then, the fixed cells were permeabilized for 5 min at room temperature with 0.3% TritonX-100 and blocked with 10% normal donkey serum in PBS for 30 min. Then, they were incubated with primary antibodies (rabbit anti-NRP1, ab81321, Abcam, 1:250; mouse anti-alpha 1 Na/K-ATPase, ab7671, Abcam, 1:250) at 4°C overnight. The cells were washed with PBS followed by incubation with the corresponding secondary antibodies (Rhodamine-conjugated goat anti-rabbit, 1:1000; AlexaFluor 488-conjugated goat anti-mouse, Life Technologies, 1:1000) for 1h at room temperature in the dark. Fluorescence images were captured with fluorescence (Olympus, IX71, Japan) or confocal laser fluorescence (Olympus, FV10i, Japan) microscopes.

### Oncomine database analysis

To identify changes in gene expression in GBM, we used the Oncomine (http://www.oncomine.org) cancer microarray database [51, 52]. To show the target genes' mRNA levels of Brain GBM vs. Normal Brain, we used ‘*Brain Glioblastoma vs. Normal Analysis’* as a filter and identified 1 brain microarray in the TGCA database with 557 samples and 12,624 measured genes (TCGA Brain, No Associated Paper, 2013). Then, we used ‘*target genes’* as a filter to explore the changes in gene expression between GBM and normal brains.

### Co-immunoprecipitation

Protein A/G-agarose beads were pre-incubated with 10 ug/ml anti-GDNF, 1:100 anti-NRP1 or IgG antibodies for 6h at 4°C. Then, membrane protein samples from C6 glioma cells treated with or without 40 ng/ml GDNF were added with the protein A/G-agarose beads and various antibodies, and rocked at 4°C overnight. Further, the beads were washed thrice with loading buffer and boiled in 1x SDS loading buffer for 1000g. After cooling to the room temperature, the samples were centrifuged (10000g) for 3min at 4°C and the supernatant was stored at -20°C. Finally, the corresponding Western blot experiments were performed using anti-GDNF or anti-NRP1 (details see “Western blot analysis” paragraph in this section).

### Molecular modeling

We identified the 3D structures of GDNF and NRP1 from the Protein Data Bank (PDB). 4UX8 is electron microscopy structure of the GDNF-GFRA1-RET complex with residues 78-211 of the D chain of GDNF [53]. 2ORX is X-ray diffraction structure of amino acid residues 273-586 of NRP1 (chain A) consisting of the F5/8 type C1 and C2 domains [54]. For molecular modeling, the D chain of 4UX8 and the A chain of 2ORX were prepared by the PyMOL Molecular Graphics System (version 0.99 Schrödinger, LLC) and the histidine residues were protonated at pH 6.5 using the PDB2PQR server 6 [55]. Then, NRP1-active sites were found with the Active Site prediction server (http://www.scfbio-iitd.res.in/dock/ActiveSite.jsp) and the largest active pocket was chosen to dock. Further, the GDNF and NRP1 proteins were docked using the ZDOCK Server [56]. Finally, the docking results were analytically visualized by the PyMOL software with all hydrogen bonds and their corresponding residues labeled. In addition, the electrostatic interactions between NRP1 and GDNF were performed by the Adaptive Poisson-Boltzmann Solver (APBS) plug-in of the PyMOL software.

### NRP1 knockdown in C6 cells by lentiviral RNAi

The RNAi for rat NRP1 (5’-GGACAGAGACTGCAAGTAT-3’) was generated with the Block-it RNAi design program (Invitrogen, Carlsbad, CA, USA), whereas the control RNAi was generated using a scrambled sequence (5’-TTCTCCGAACGTGTCACGT-3’) ensuring no homology with rat genome. The RNAi sequences were cloned into GV248 (GeneChem, Shanghai, China) to generate lentiviral shRNA vectors. Both the expression vectors and package vectors were then transfected into 293T cells by Lipo2000 (Invitrogen, USA). The lentivirus containing supernatants were harvested, denoted as GV248-NRP1-shRNA-LV and CON077-negative-shRNA-LV, respectively. The lentiviruses were concentrated by ultracentrifugation and their titers were determined (LV-NRP1-RNAi, 5e^8^ TU/ml; CON077-negative-shRNA-LV, 1e^9^ TU/ml).

For lentivirus infections, the C6 glioma cells (4×10^3^ cells/well) were cultured overnight in 96-well microplates. The diluted lentiviruses in 0.2ml complete medium with 10 μg/ml polybrene were added to C6 cells and cultured for 12h at 37°C. The medium containing virus was replaced with fresh medium. Successfully infected cells were identified by observing GFP expression in a fluorescent microscope (Olympus, IX71, Japan).

### Western blot analysis

After blocking by 5% skimmed milk, the samples were incubated with primary antibody (rabbit anti-NRP1, ab81321, Abcam, 1:250; mouse anti-alpha 1 Na/K-ATPase, ab7671, Abcam, 1:250; rabbit anti-GDNF, ab18956, Abcam, 1:100; mouse anti-β-actin, sc47778, Santa cruz, 1:1000; rabbit anti-Histone H3 antibody, BS1660, bioworld, 1:500) at 4°C overnight [57]. Then, the samples were incubated with IRdye secondary antibodies (goat anti-Rabbit, LI-COR, Odyssey, 1:1000; goat anti-Mouse, LI-COR, Odyssey, 1:1000) at room temperature for 2h. Finally, the protein bands were scanned by Odyssey imaging system (LI-COR, USA) and quantified with ImageJ software (National Institutes of Health, USA).

## QRT-PCR

Total RNA was isolated from C6 glioma cells and its concentration and quality was determined with Nanodrop ND-1000 spectrophotometer (NanoDrop Technologies, Wilmington, DE, USA) and gel analysis. Then, the RNA samples were reverse transcribed to cDNA using Transcriptor First Strand cDNA synthesis kit (Roche Applied Science, Penzberg, Germany). Then, real time PCR was performed to analyze the expression of NRP1 using the SYBR Green PCR master mix (Roche Applied Science, Mannheim, Germany). The 20 μl reaction mixture included 10 μl 2×SYBR^®^ Premix Ex Taq (Takara, China),0.5 μl each of 10 μM forward and reverse primers, 2 μL cDNA template and 7 μL RNase-free H_2_O. The PCR conditions included initial denaturation at 95°C for 5 min followed by 45 cycles of 95°C for 10 sec, 60°C for 15 sec and 72°C for 15 sec.

The mRNA data were normalised to β-actin. The primers for NRP1 and β-actin were as follows:

β-actin: Forward: 5-AGCCATGTACGTAGCCATCCA-3
 Reverse: 5-TCTCCGGAGTCCATCACAATG-3
NRP1: Forward: 5-GGAGCTACTGGGCTGTGAAG-3
 Reverse: 5-ATGTCGGGAACTCTGATTGG-3

### Survival analysis

The dataset of Glioblastoma Multiforme was obtained via cBioportal from the TCGA website (http://cancergenome.nih.gov) [58, 59]. Then, median expression of the mRNAs was determined (RNA Seq V2 RSEM). Patients were divided into 2 groups, namely, high NRP1 expression group and the control group. The two-sample rank-sum test and multivariate proportional hazard model analysis were performed with the overall survival (OS) and disease-free survival (DFS) data of the GBM patients and Kaplan-Meier curves were constructed.

### Statistical analysis

Statistical analysis was performed using SPSS 17.0 for Windows. The data were presented as mean ± standard deviation obtained from three independent experiments. One-way ANOVA and Tukey's post-hoc analysis were used to determine the differences between groups. *P*<0.05 was considered statistically significant.

## Abbreviations

GBM: Glioblastoma
GDNF: Glial cell line-derived neurotrophic factor
NRP1: neuropilin-1
CNS: central nervous system
GFL: GDNF family of ligands
GFRA1: GDNF family receptor alpha 1
RET: receptor tyrosine kinase
NCAM: neural cell adhesion molecule
PPIs: protein-protein interactions
LC-MS/MS: liquid chromatography-tandem mass spectrometry
GO: Gene Ontology
DMEM/F-12: Dulbecco's Modified Eagle Medium/Nutrient Mixture F-12
FBS: fetal bovine serum
SD: Sprague-Dawley
GST: glutathione S-transferase
NTDK: Neural Tissue Dissociation Kits
GFAP: glial fibrillary acidic protein
FASP: filter aided sample preparation
PI: propidium iodide
CCK8 assay: cell counting kit-8 assay
OD values: optical density values
EdU: 5-ethynyl-2′-deoxyuridine
SDS-PAGE: sodium salt-Polyacrylamide gel electrophoresis
ESI: electrospray ionization
PANTHER: Protein ANalysis THrough Evolutionary Relationships
PDB: Protein Data Bank
LV: lentivirus
AST: primary astrocytes
ITGB1: Integrin Subunit Beta1
LFQ: Label-free quantitative
CDH2: Cadherin-2
SDC3: Syndecan-3
ATRN: Attractin
IF: immunofluorescent
OS: overall survival
DFS: disease-free survival
Sema3A: semaphorin3A
DAs: dopaminergic neurons
DEPs: differentially expressed proteins
Co-IP: Co-Immunoprecipitation
VEGF: Vascular Endothelial Growth Factor
VEGFR: Vascular Endothelial Growth Factor Receptor
DAPI: 4',6-diamidino-2-phenylindole
FC: Fold Change
